# Resistance of Gram-Negative Bacteria to Current Antibacterial Agents and Approaches to Resolve It

**DOI:** 10.3390/molecules25061340

**Published:** 2020-03-16

**Authors:** Zeinab Breijyeh, Buthaina Jubeh, Rafik Karaman

**Affiliations:** Department of Bioorganic & Pharmaceutical Chemistry, Faculty of Pharmacy, Al-Quds University, Jerusalem P.O. Box 20002, Palestine; z88breijyeh@gmail.com (Z.B.); bjubeh@gmail.com (B.J.)

**Keywords:** antimicrobial, antibiotic, resistance, Gram-negative, multidrug resistance (MDR), pathogens, bacteria, alternative therapies

## Abstract

Antimicrobial resistance represents an enormous global health crisis and one of the most serious threats humans face today. Some bacterial strains have acquired resistance to nearly all antibiotics. Therefore, new antibacterial agents are crucially needed to overcome resistant bacteria. In 2017, the World Health Organization (WHO) has published a list of antibiotic-resistant priority pathogens, pathogens which present a great threat to humans and to which new antibiotics are urgently needed the list is categorized according to the urgency of need for new antibiotics as critical, high, and medium priority, in order to guide and promote research and development of new antibiotics. The majority of the WHO list is Gram-negative bacterial pathogens. Due to their distinctive structure, Gram-negative bacteria are more resistant than Gram-positive bacteria, and cause significant morbidity and mortality worldwide. Several strategies have been reported to fight and control resistant Gram-negative bacteria, like the development of antimicrobial auxiliary agents, structural modification of existing antibiotics, and research into and the study of chemical structures with new mechanisms of action and novel targets that resistant bacteria are sensitive to. Research efforts have been made to meet the urgent need for new treatments; some have succeeded to yield activity against resistant Gram-negative bacteria by deactivating the mechanism of resistance, like the action of the β-lactamase Inhibitor antibiotic adjuvants. Another promising trend was by referring to nature to develop naturally derived agents with antibacterial activity on novel targets, agents such as bacteriophages, DCAP(2-((3-(3,6-dichloro-9*H*-carbazol-9-yl)-2-hydroxypropyl)amino)-2(hydroxymethyl)propane1,3-diol, Odilorhabdins (ODLs), peptidic benzimidazoles, quorum sensing (QS) inhibitors, and metal-based antibacterial agents.

## 1. Introduction

Throughout history, natural products have been utilized to treat a variety of diseases; cinchona tree containing quinine to treat malaria, penicillin for the treatment of infectious diseases, and others. Since the discovery of penicillin by Fleming in 1929, a large number of antibacterial agents have been developed and have had a huge impact on human health and the mortality rates of humans around the world [1].

Widespread excessive dispensing and irresponsible use of antibiotics has resulted in the development of resistant strains. Unfortunately, most antibiotics are available over the counter in the developing countries and can be dispensed without prescription; therefore, patients and general public education are crucially needed [2].

The global effort to develop new antibiotics or modify existing ones to fight resistant pathogens globally is now huge. Antibiotic resistance evolves when the bacteria can escape the effect of antibiotics by different mechanisms, like neutralizing the antibiotics, pumping them outside of the cell, or modifying their outer structure resulting in inhibition of the drugs’ attachment to the bacteria. The mechanisms of antibiotic resistance are categorized into four groups: intrinsic resistance in which bacteria can change their structures or components, another way is acquired resistance, where bacteria can acquire new resistance genes and DNA from other resistant bacteria. Furthermore, genetic changes in the DNA which can alter the production of protein leading to different components and receptors that cannot be recognized by the antibiotic, and finally DNA transfer through a horizontal gene transfer between bacteria *via* transformation or transduction or by conjugation [3].

**Figure 1:**
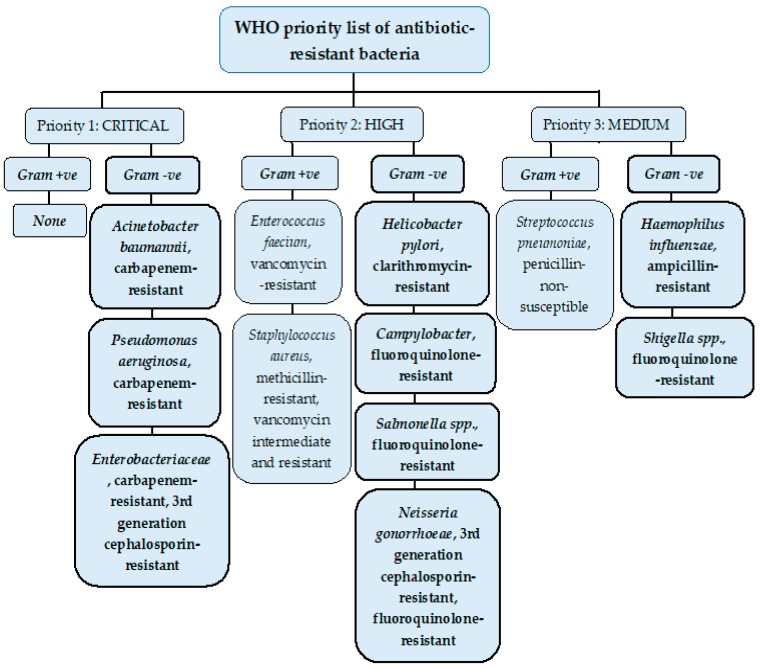
WHO list of priority pathogens grouped under three priority categories according to their antibiotic resistance: Critical, high and medium to encourage research and development of new antibiotics.

Resistance to antimicrobials is a growing crisis in clinical medicine. In 2017, the WHO published a list of bacteria where new antibiotics to tackle them are needed urgently and grouped them according to their priority as critical, high, and medium (Figure 1).

### Gram Negative Bacteria

In 1884 Hans Christian Gram developed a method to distinguish between Gram-positive and Gram-negative bacteria by using a crystal violet-iodine complex and a safranin counter stain. Gram- positive bacteria stained violet or purple and Gram-negative bacteria don’t retain the complex stain and counter stain with safranin to give a pink color. This difference is due to the composition or the morphology of the cell wall in each bacterial type [1,2].

**Figure 2:**
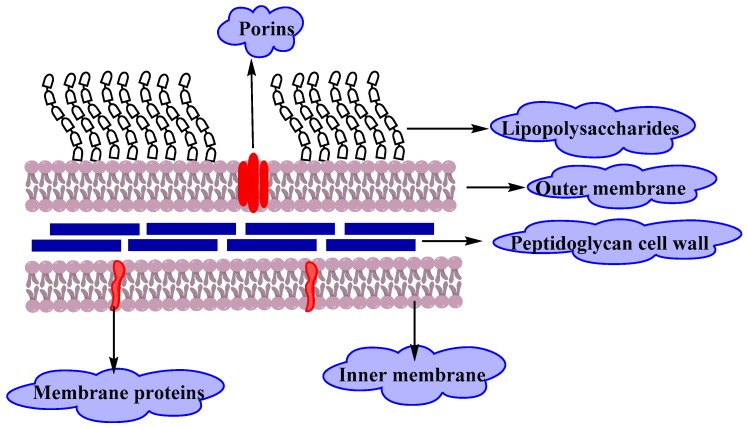
A diagram describes the cell wall structure of Gram-negative bacteria.

Gram-negative bacteria have an envelope that consists of three layers (Figure 2).The first layer is the outer membrane (OM), a protective and a unique feature that distinguishes Gram-negative bacteria from Gram-positive bacteria. The OM has phospholipids that are bound to the inner leaflet of the membrane, and lipopolysaccharide (LPS) bound to the outer leaflet which is known to cause endotoxic shock. Moreover, the OM contains proteins called the outer membrane proteins (OMPs) such as porins and others which allow the passage of small molecules like amino acids and small saccharides. The second layer is the peptidoglycan cell wall which is a rigid exoskeleton that determines the cell shape and consists of a repeat unit of the disaccharide N-acetyl glucosamine-N-acetylmuramic acid [3]. The third layer is the inner membrane (IM) which is a phospholipid bilayer that is responsible for multifunctional processes like structure, transport, and biosynthetic functions. In addition, it is the site for DNA anchoring and plays an important role in sister chromosomes separation [4].

The outer membrane of Gram-negative bacteria is the main reason for resistance to a wide range of antibiotics including β-lactams, quinilons, colistins and other antibiotics. Most antibiotics must pass the outer membrane to access their targets, for example, hydrophobic drugs can pass through by a diffusion pathway, on the other hand, hydrophilic antibiotics like β-lactams pass through porins, and vancomycin can’t cross the outer membrane due to its structure that hinder it from using any of these passages. Any alteration in the outer membrane by Gram-negative bacteria like changing the hydrophobic properties or mutations in porins and other factors, can create resistance. Gram-positive bacteria lack this important layer, which makes Gram-negative bacteria more resistant to antibiotics than Gram-positive ones [5,6,7].

Gram-negative bacteria can cause serious diseases in humans, especially in immuno-compromised individuals. Nosocomial infections caused by Gram-negative bacilli (GNB) are the most challenging issue for health care professionals due to resistance to antibiotics [8].

Resistant GNB is responsible for most of the cases of ventilator-associated pneumonia, catheter-related bloodstream infections and other ICU-acquired sepsis such as urinary tract infections. The major Gram-negative bacteria that cause complications are *Enterobacteriaceae* and non-fermenting GNB (*Pseudomonas aeruginosa, Acinetobacter baumannii* and *Stenotrophomonas maltophilia*).

**Figure 3:**
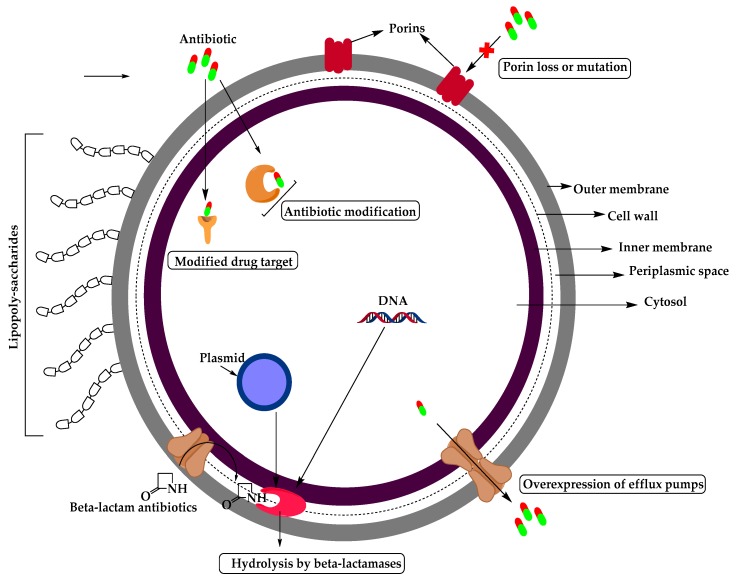
Structure of Gram-negative bacteria and their mechanisms of resistance.

The mechanism of antimicrobial resistance in GNB arises from the expression of antibiotic inactivating enzymes and non-enzymatic paths (Figure 3) which may result from increasing the intrinsic resistance due to mutations in chromosomal genes (such as increasing the expression of antibiotic-inactivating enzymes, efflux pumps, permeability or target modifications) or acquired by transfer of mobile genetic elements carrying resistance genes such as plasmid encoding β-lactamases, aminoglycosides modifying enzymes, or non-enzymatic mechanisms like Qnr (plasmid-borne quinolone resistance gene) for fluoroquinolone (FQ) resistance in *Enterobacteriaceae* [9].

*Enterobacteriaceae* resistance to third generation cephalosporins is now above 10%, and 2-7% for carbapenem. This is because of the rapid spread of extended-spectrum β-lactamase (ESBL) producing strains. Carbapenem resistance rates for *klebsiella* *pneumonia* are above 25% while 20 to 40% is for *P. aeruginosa* and 40 to 70% ICU acquired infections being carbapenem-resistant for *A. baumannii* [9].In this review, we discuss the most important resistant Gram-negative bacteria at a global level as determined by the WHO, and the treatment approaches to combat such resistance.

## 2. Resistant Gram-Negative Bacteria

### 2.1. Enterobacteriaceae

*Enterobacteriaceae* family such as *Escherichia coli, Klebsiell* spp., and *Enterobacter* spp. is the major cause of urinary tract infections (UTIs), blood-stream infections, hospital, and healthcare-associated pneumonia. Resistance is mainly related to the production of ESBLs, but other mechanisms of resistance are also emerging, leading to multidrug-resistance (MDR) [10].

#### 2.1.1. Enterobacteriaceae- 3rd Generation Cephalosporin-Resistant

*Enterobacteriaceae* resistance to third-generation cephalosporins is a result of the production of β-lactamases. For example, ESBLs can hydrolyze broad-spectrum cephalosporins, monobactams, and penicillins. Enzymes of class A β-lactamases, like TEM-1, TEM-2, and SHV-1 are responsible for the resistance to ampicillin, amoxicillin, and early generation cephalosporins. Resistance to third-generation cephalosporins arises when mutation of genes encoding TEM-1, TEM-2, or SHV-1 gives rise to new β-lactamases that can hydrolyze them.

Other types of ESBLs may be expressed by *Enterobacteriaceae* like, CTX-M (CTX-Munich, an ESBL enzyme) that hydrolyzes cefotaxime more efficiently than ceftazidime and carbapenem hydrolyzing oxacillinases (OXA) which are mainly found in *P. aeruginosa* and rarely in *Enterobacteriaceae*. In addition to ESBLs, AmpC β-lactamases are also able to hydrolyze third-generation cephalosporins and are resistant to inhibition by clavulanate and other β-lactamase inhibitors [10].

#### 2.1.2. Enterobacteriaceae- Carbapenem-Resistant

Carbapenem-resistant *Enterobacteriaceae* (CRE) is an *Enterobacteriaceae* isolate that is resistant to ertapenem, imipenem, meropenem or any carbapenem antimicrobial. The first isolates were reported in the 1990s and the resistance was due to AmpC β-lactamase production and loss of outer membrane protein. There are two types of CRE: carbapenemase-producing CRE (CP-CRE) in which their genes are present on mobile genetic elements and none carbapenemase-producing CRE (non-CP-CRE) [11].

There are five major carbapenemases which include: (1) *Klebsiella pneumonia carbapenemase*(KPC), class A serine based β-lactamases, (2) class B, New Delhi Metallo-β-lactamases (NDM), (3) Verona integrin encoded Metallo-β-lactamase (VIM), (4) class D, OXA or OXA-48-like carbapenemases and (5) IMP, active on imipenem. *Enterobacteriaceae* species that have intrinsic imipenem resistance include *Morganella morganii, Proteus* spp. And *Providencia* spp. [12]

### 2.2. Acinetobacter baumannii

*Acinetobacter baumannii* is an aerobic Gram-negative bacteria and one of the most serious *Enterococcus faecium, Staphylococcus aureus, Klebsiella pneumonia, A. baumannii, Pseudomonas aeruginosa,* and *Enterobacter* species (ESKAPE) organisms, as declared by the WHO that can escape the effect of antibacterial drugs [13]. *A. baumannii* is associated with hospital-acquired infections worldwide and rapidly develops resistance to antimicrobials by different mechanisms such as:

(1) The inactivation of β-lactams by β-lactamases which is considered as a major MDR mechanism in *A. baumannii*. All four classes of β-lactamases; A, B, C, and D were identified in *A. baumannii*, which can incorporate exogenous DNA into its genome and identify a large number of β-lactamases. Some of these enzymes are narrow-spectrum β-lactamases like TEM-1, SCO-1, and CARB-4, but some others are responsible for the hydrolysis of ESBL; GES-11 and CTX-M which can reduce susceptibility to carbapenems. Class B is metallo-β-lactamases (MBLs) that have a broad range, potent carbapenemase activity and resistance to all β-lactam antibiotics but not to monobactams. Class C β-lactamases are resistant to cephamycins (cefoxitin and cefotetan), penicillins and cephalosporins. *A. baumannii* has an intrinsic AmpC cephalosporinase. Class D or OXAs β-lactamases preferred to oxacillin can hydrolyze extended-spectrum cephalosporins and carbapenems.

(2) Another resistance way is multidrug efflux pumps against many different classes of antibiotics, including tigecycline or imipenem resistance in *A. baumannii*. There are four categories of efflux pumps: the resistance nodulation division (RND) superfamily, the major facilitator superfamily (MFS), the multidrug and toxic compound extrusion (MATE) family and the small multidrug resistance (SMR) family transporters. Overexpression of the AdeABC efflux pump an RND type results in decreasing susceptibility to tigecycline.

(3) The resistance of *A. baumannii* to aminoglycoside is mediated by three classes of enzymes, including acetyltransferases, adenyltransferases, and phosphotransferases. These enzymes chemically modify aminoglycosides. The coding genes can be transferred through plasmids, transposons, and integrons.

(4) Permeability defects by changing in envelope permeability. Porins are proteins that form channels to allow transport of molecules across the outer membrane and play a significant role in the mechanism of resistance. Reducing the expression of some porins like Caro, Omp22-33 is associated with carbapenem resistance in *A. baumannii*. In addition to outer membrane proteins, loss of LPS increases colistin resistance in *A. baumannii* due to a decrease in membrane integrity.

(5) Alteration of target sites, such as penicillin-binding proteins (PBPs), mutations of DNA gyrase and others, alter the target sites for antibiotics. Overexpression of certain PBPs results in imipenem resistance and mutation in DNA gyrase as in the cases of quinolone and tetracycline resistance in *A. baumannii*.

(6) Integrons are located on bacterial chromosomes or plasmids and have four classes. Integrons have a unique capacity to cluster and express drug resistance genes and are a useful marker for epidemic strains of *A. Baumannii* [13,14].

Carbapenems like imipenem, meropenem, and doripenem were the best agents to treat*A. baumannii,* but due to the decreased susceptibility of *A. baumannii* to those agents, minocycline/tigecycline and polymyxins are the most effective and showed a synergistic effect against *A. baumannii* infections. However, tigecycline and colistin-resistant *A. baumannii* arose. The combined therapy of ampicillin with sulbactam and ampicillin + sulbactam + carbapenem combination therapy is effective for treating MDR *A. baumannii* bacteremia. Minocycline therapy also has high treatment success rates and good tolerability, but due to the introduction of minocycline, around 20% of *A. baumannii* have developed resistance. Minocycline combined with colistin is effective for treating minocycline-resistant *A. baumannii* infections and colistin/rifampin is the most effective treatment for colistin-resistant *A. Baumannii.* Also, trimethoprim-sulfamethoxazole combined with colistin rapidly kills carbapenem-resistant *A. baumannii* infections. Other non-antibiotic therapies are bacteriophages which are viruses that lyse the bacteria. American alligator plasma peptide and antimicrobial peptide dendrimer G3KL have in vitro antimicrobial activity against MDR *A. baumannii* but they have a short half-life and high production costs. Therefore, effort must be done to invoke new strategies for discovering new classes of antibiotics to control *A. baumannii* infections successfully [13,14].

### 2.3. Pseudomonas aeruginosa

*Pseudomonas aeruginosa* is a Gram-negative aerobic bacterium found as part of normal intestinal flora and a powerful pathogen classified as an ESKAPE organism responsible for ICU-acquired infections in critically ill patients. Many mechanisms can contribute to its antibiotic resistance: innate resistance of *P. aeruginosa* such as over-expression of efflux pumps and decreasing outer membrane permeability, also acquired resistance mechanisms like acquisition of resistance genes or mutation in genes that encode for porins and other proteins these all can make this microorganism difficult to treat.

*P. aeruginosa* was first isolated from green pus in 1882 and found to be an opportunistic pathogen in immuno-compromised patients that can survive on dry surfaces of hospital environments such as respiratory equipment and dialysis tubing. It is the fourth-most commonly isolated nosocomial pathogen, the second-most common cause of ventilator-associated pneumonia and the third-most common Gram-negative cause of bloodstream infections.

β-Lactam antibiotics such as penicillin, cephalosporin, and carbapenem inhibit the synthesis of bacterial peptidoglycan cell walls. The third and fourth generation of cephalosporins like ceftazidime and cefepime, respectively, are the most effective β-lactams used in the treatment of *P. aeruginosa*. Resistance to these antibiotics is mediated by β-lactamases which destroy the amide bond of the β-lactam ring and make the antibiotics ineffective. There are four major classes of β-lactamases that have been identified in *P. aeruginosa*: Classes A, C, and D inactivate the β-lactams through the catalytic activity of serine-residue, whereas class B or MBLs need zinc cation for their action.

Endogenous β-lactamase such as AmpC β-lactamase can be induced by several β-lactams such as benzylpenicillin and imipenem. *P. aeruginosa* can acquire resistance through a gene mutation which leads to overexpression of AmpC β-lactamase. Several genes are involved in the induction of ampC gene including ampR that encodes for a positive transcriptional regulator which is necessary for β-lactamase induction, ampG which encodes a transmembrane protein that acts as a permease for 1,6-anhydromurapeptides that induce ampC, and ampD which encodes a cytosolic N-acetyl-anhydromuramyl-L-alanine amidase and acts as a repressor of ampC expression. AmpE is the fourth gene that encodes a cytoplasmic-membrane protein that acts as a sensory transducer molecule necessary for induction.

*Pseudomonas* resistance to aminoglycosides is mediated by transferable aminoglycoside modifying enzymes (AMEs) which are divided into three classes: aminoglycoside phosphoryl transferases (APHs), aminoglycoside adenylyl transferases (AADs) and aminoglycoside acetyltransferases (AACs) that inactivate aminoglycoside by attaching a radical of phosphate, adenyl or acetyl to antibiotic molecule, and decrease the binding affinity to their target in the bacterial cell.

Resistance to FQ is developed via mutation in the bacterial chromosomal gene encoding DNA gyrase or topoisomerase 1 V or by active transport of a drug out of the cell.

Colistin found to be effective in treating MDR *Pseudomonas* than otherβ-lactam drugs and was more efficient when used in combination with an anti-pseudomonas agent like imipenem, piperacillin, aztreonam, ceftazidime or ciprofloxacin. Also, fosfomycin therapy with aminoglycosides, cephalosporins, and penicillins, has been used for a better result for the treatment of drug resistance *P. aeruginosa* [15].

### 2.4. Helicobacter pylori- Clarithromycin-Resistant

*Helicobacter pylorus* (HP) is a Gram-negative bacterium recognized as the most important pathogen responsible for infections in humans such as gastritis, peptic ulcers and gastric cancer. The efficacy of the treatment of HP has decreased due to the quick development of antibiotic resistance [16,17]. Clarithromycin is part of the first-line triple therapy; resistance may develop due to different mutations in the domain V of the 23S rRNA gene such as *A2142G, A2142C, or A2143G* in the bacteria which results in decreasing the affinity to the drug. Other resistances also were reported which were linked to translation initiation factor IF-2, ribosomal protein L22 and overexpression of efflux pumps. According to the WHO, clarithromycin resistance to HP is listed as a high priority for antibiotic research and development [18].

### 2.5. Campylobacter- Fluoroquinolone-Resistant

*Campylobacter* are Gram-negative bacteria cause infection to human and animals such as gastroenteritis usually after consuming contaminated or undercooked food. *Campylobacter jejuni* and *Campylobacter coli* are common pathogenic species that colonize several animal foods like poultry. No antibiotics are required for treatment because it is usually self-limiting but in severe cases, FQ such as ciprofloxacin is used. In the 1980s, FQ resistance *Campylobacter* was reported and spread rapidly and was due to independent mutation and horizontal transfer of resistance DNA among strains, single point mutation C257T in the *gyrA* gene has been reported in ciprofloxacin resistance strains which results in an amino acid substitution in the gyrase A subunit. Resistance to FQ can be enhanced by overexpression of *the Cme*ABC efflux pump, a mutation in 16 bp inverted repeat (IR) in the *cme*R–*cme*ABC intergenic region and variation if mutant frequency decline gene (*mfd*) [19,20].

### 2.6. Salmonella spp.- Fluoroquinolone-Resistant

*Salmonellae* are Gram-negative bacteria, subdivided into two groups: typhoidal *Salmonella* and non-typhoidal *Salmonella* (NTS) which are pathogenic to humans. MDR in *Salmonella* to ampicillin, chloramphenicol and trimethoprim/sulfamethoxazole led to the intensive use of FQ ciprofloxacin and the third-generation cephalosporin ceftriaxone which rapidly led to the development of resistance to these drugs. This was the reason behind the ranking of FQ-resistant *Salmonella* as a high priority pathogen for the research and development of new antibiotics by the WHO in 2017.

Resistance to quinolones is a result of multiple mechanisms such as mutations in the quinolone resistance determining regions (QRDRs) of the chromosomal *gyr* and *par* genes which result in low binding affinity of quinolone to topoisomerase enzymes. Another mechanism is plasmid-mediated quinolone resistance (PMQR) like *Qnr* genes which provide physical protection from quinolones, the *aac(6′)-lb-cr*gene decreases FQ activity, and *oqxAB* and *qepA* encodes quinolone efflux pumps [21].

#### 2.6.1. Typhoidal Salmonella Resistance

In the seventies, chloramphenicol was the treatment of choice for enteric fever caused by Salmonella Typhi, but chloramphenicol-resistant strain starts to appear due to determinant located on a self-transmissible plasmid of the HI1 incompatibility type (IncHI). This led to an increase in the use of ampicillin and trimethoprim-sulfamethoxazole to the 1980s when their resistance was reported from multiple countries. Ciprofloxacin was used as an alternative treatment after the spread of MDR Salmonella Typhi in 1992 [22].

#### 2.6.2. Non-typhoidal Salmonella Resistance

In the eighties, MDR Salmonella Typhimurium began to appear and was associated with a phage-type called definitive type 104 (DT104). The isolates are resistant to ampicillin, chloramphenicol, streptomycin, sulfonamides, and tetracycline. FQ resistance was developed among non-typhoidal Salmonella after the introduction of FQs as an alternative treatment [22].

### 2.7. Neisseria gonorrhoeae

*Neisseria gonorrhoeae* is a Gram-negative diplococcus and an obligatory human pathogen responsible for the sexually transmitted disease gonorrhea. The gonococcus can infect different mucosal surfaces such as urethra, endo-cervix, pharynx, conjunctiva and the rectum [23]. The spread of gonococcal infections is due to the ability of *N. gonorrhea* to acquire resistance to antibiotics like penicillin, tetracycline, and quinolones. In 1936 sulfonamides were the best treatment for gonococcal but resistance developed shortly thereafter. In 1940s penicillin was introduced and penicillinase-producing *N. gonorrhoeae* (PPNG) was spread, which led to a switch to alternative therapy. Tetracyclines were widely used in some developing countries and the first reports of tetracycline resistant *N. gonorrhea e*(TRNG) appeared in 1985 [24].

#### 2.7.1. Neisseria gonorrhoeae- 3rd Generation Cephalosporin-Resistant

Third-generation cephalosporins have broader activity against Gram-negative bacteria and are used frequently to treat *N. gonorrhoeae*. Resistance to cephalosporins started to develop and spread in Asia and the United States in the 1990s then to Australia and Europe in the 2000s.There are several mechanisms for cephalosporins resistance: (1) altered PBPs; *N. gonorrhoeae* has three penicillin-binding proteins (PBPs), alteration in PBP2 by the *penA* gene results in decreasing the binding of penicillin through a single amino acid insertion (Asp-345a). This alteration is mostly related to cephalosporin resistance, (2) changes in *penA* transpeptidase domain to form mosaic *penA*; responsible for most observed reduced susceptibility to cephalosporins such as cefixime. (3) A reduction of intracellular antimicrobial concentration by preventing its entry or actively pumping by efflux pumps like the MtrC-D-E system, a mutation in the *mtrR* gene which results in increasing efflux and resistance to antibiotics. Finally, (4) mutations in the *penB* porin gene reduce permeability to antimicrobial agents [25].

#### 2.7.2. Neisseria gonorrhoeae- Fluoroquinolone-Resistant

Quinolones affect the activity of DNA gyrase and topoisomerase IV, resistance to ciprofloxacin is mediated by mutations in quinolone resistance determining region (QRDR), single or more mutation in amino acids in gyrA positions 91, 95 and 102, and point mutation in parC genes which code for the DNA gyrase and topoisomerase IV proteins led to increasing resistance to ciprofloxacin and prevented it from binding to their target enzymes. Other mechanisms of FQ resistance include overexpression of efflux pumps like *NorM* pumps and decrease the permeability of antimicrobial agents by a reduction in the outer membrane porin protein expression [26,27].

### 2.8. Haemophilus influenza- Ampicillin-resistant

*Haemophilus influenza* are Gram-negative, coccobacilli, facultatively anaerobic bacteria that have two types based on its polysaccharide: (1) capsulated with six serotypes from ***a*** to ***f*** or (2) non-capsulated [28]. Pneumonia, meningitis, and bacteremia are the major diseases caused by type b strain *H. influenza* while community-acquired pneumonia, acute otitis media, and sinusitis are commonly caused by a non-capsulated form [29]. 

Ampicillin, which inhibits the synthesis of the cell wall, is the main treatment against *H. influenza*. The mechanism of resistance is either by acquired β- lactamases or by PBP target modifications or efflux mechanisms [28]. Resistance to ampicillin is mediated by the production of the β-lactamase TEM-1 or ROB-1 (TEM-1 and ROB-1 are two β-lactamases identified in *Haemophilus influenza*), which causes a decrease in the affinity of penicillin-binding proteins. *H. Influenza* isolates are classified according to their ampicillin resistance mechanism into a β-lactamase negative, ampicillin-sensitive (BLNAS) strains, β-lactamase positive, ampicillin-resistant (BLPAR) strains, BLNAR strains, and BLPACR strains.

Resistance in BLNAR strains are due to PBP 3 amino acid substitutions (as a result of an acquisition of point mutations in the *ftsI* gene by antibiotic pressure) and are classified into three groups (I, II, and III) according to Ubukata et al. and Dabernat et al., however, BLPACR are more resistance to amoxicillin-clavulanate, chloramphenicol, and cefuroxime than BLNAS strains and all strains of BLPACR had TEM-1 type β-lactamase and multiple mutations within the *ftsI* gene [30]. High levels of resistance among *H. Influenza* encouraged the WHO to recognize it in the list of medium-priority antimicrobial-resistant pathogens.

### 2.9. Shigella spp.- Fluoroquinolone-Resistant

*Shigella seps* is a Gram-negative bacterium including four types: *Shigella flexneri* (the most predominant), *Shigella sonnei*, *Shigella boydii*, and *Shigella dysenteriae. Shigella* infection causes acute dysentery or chronic diarrhea.

FQs are the best treatment for *Shigella* infection but antimicrobial resistance reduces the effectiveness of this antibiotic. FQ resistance is associated with multiple mutations in the quinolone resistance determining region (QRDR) that encodes DNA gyrase (gyrA and gyrB) and topoisomerase IV (parC and parE). This is in addition to plasmid-mediated quinolone resistance due toQnr genes and efflux pump mediators like mdfA, tolC, ydhE and marA [31,32].

## 3. Treatment

Antimicrobial resistance to antibiotics is a huge and growing concern. Antimicrobial resistances estimated to cause more than 700 thousand deaths annually worldwide and this number is expected to grow to 10 million by the year 2050, according to the U.S. Centers for Disease Control and Prevention (CDC). New antimicrobial drug development is needed urgently and is considered as a high priority. Recent developments are now focusing on natural products and the return of natural product screening to discover new therapeutics to fight resistant pathogens. Herein, we discuss some new novel treatments that have emerged from research and development programs to be used against resistant Gram-negative bacteria [33].

### 3.1. Antibiotic Adjuvants

Antibiotic adjuvants, also called ‘resistance breakers’ or ‘antibiotic potentiators’, are compounds that have no antibiotic activity, or very little antibiotic activity; but when administered with antibiotics as a combined drug therapy, they enhance the activity of the drug or block the resistance of the bacteria toward that drug [34,35]. Antibiotic adjuvants revived the use of some antibiotics against resistant pathogens; this lessens the urge to discover new antibiotics with novel targets, which is an expensive and challenging mission. 

Three types of antibiotic adjuvants have been developed (or are being developed) so far: the β-lactamase inhibitors, inhibitors of the efflux pump, and outer membrane permeabilizers. These adjuvants help antibiotics overcome bacterial resistance which happens in one of four mechanisms: enzyme inactivation of the drug, drug efflux by efflux pumps, uptake decrease as a result of membrane permeability change, and drug target modifications [36]. In this review, we will focus on β-lactamase inhibitors.

#### 3.1.1. β-Lactamase Inhibitors

β-Lactamase inhibitors are the most clinically used antibiotic adjuvants. They are used to overcome the resistance to β-lactam antibiotics. β-lactamase inhibitors remained the most successful antibiotic adjuvants despite their long-term use (more than 70 years) [37].

**Figure 4:**
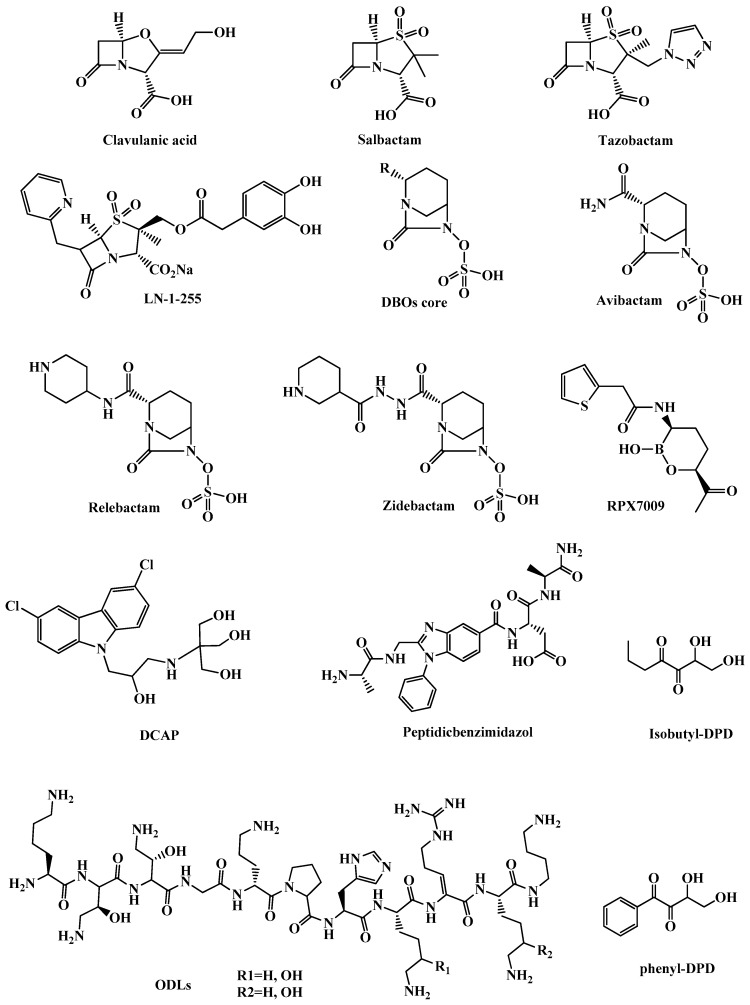
Chemical structures of clavulanic acid, salbactam, tazobactam, LN-1-255, DBOs core, relebactam, zidebactam, RPX7009, DCAP, peptidic benzimidazol, isobutyl-DPD and phenyl-DPD.

β-Lactamases are enzymes produced by resistant bacteria that hydrolyze the β-lactam core of β-lactam antibiotics through an acylation-deacylation-based process. They are categorized into two groups: (1) Serine-β-lactamases in which a nucleophilic serine moiety of the lactamase binds covalently to a hydrolyzed β-lactam, this group is subcategorized to Ambler class A, C, or D; classes that are inhibited by clavulanic acid (Figure 4), sulbactam (Figure 4), and tazobactam (Figure 4). (2) MBLs which represent Ambler class B and have active sites with one or two zinc ions that do a nucleophilic attack to the β-lactams via a polarized water molecule [35,37,38,39]. MBLs are subcategorized to B1, B2, and B3 classes according to the number of bound zinc ions and the sequence identity. Except for monobactams, MBLs are active against all β-lactam antibiotics. So far, there are no approved inhibitors for class B MBLs [40].

The most diverse and rapidly growing group of β-lactamases is carbapenem-hydrolyzing class D β-lactamases (CHDLs), also known as OXAs, it is a group of over 500 reported enzymes found among the most clinically challenging species including *A. baumannii, P. aeruginosa, E. coli, and P. mirabilis*. CHDLs can hydrolyze penicillins, cephalosporins, and aztreonam. OXA-23, OXA-24/40 and OXA-48 are the most worrisome CHDLs in clinical settings [37,39].

#### 3.1.2. Clavulanic Acid and Penicillin-based Sulfones

Clavulanic acid (Figure 4) is a β-lactam compound isolated from *Streptomyces clavuligerus* bacteria in 1976 and it was the first agent to be used as a β-lactamase inhibitor in combination with amoxicillin [41]. Clavulanic acid is structurally similar to penicillin, and it covalently binds to β-lactamase through the catalytic serine and makes a stable adduct. Clavulanic acid is clinically ineffective against class B, C, and D β-lactamases [37].

Sulbactam and tazobactam (Figure 4) are penicillin-like sulfones that have a mechanism of β-lactamase inhibition similar to that of clavulanic acid, but they undergo a ring-opening after the catalytic serine attack as a result of sulfonate group formation. Extra electrostatic interactions with the active site and generation of a good leaving group at the five-membered ring made clavulanic acid, sulbactam, and tazobactam capable of efficient irreversible inhibition of β-lactamase of class A [35,37].

Clavulanic acid, sulbactam, and tazobactam are the main β-lactamase inhibitors in clinical practice. Available combinations of β-Lactam– β-lactamase inhibitor include amoxicillin-clavulanate, ticarcillin-clavulanate, ampicillin-sulbactam, piperacillin-tazobactam, and cefoperazone-sulbactam. The latter is only used in some European countries, Japan, and India [42].

LN-1-255 (Figure 4) is a 6-alkylidene-2′-substituted penicillanic acid sulfone synthesized by Buynak and coworkers among other compounds in a search for new OXA β-lactamase inhibitors [43]. LN-1-255 had in vitro activity against OXA, a clinically important β-lactamase (CHDL class) found in *A. baumannii* that inactivates carbapenems. LN-1-255′s efficacy of inhibition was 10-1000 folds higher than tazobactam and avibactam. LN-1-255 has the potential to be a new treatment for resistant *A. baumannii* strains combined with carbapenems or cephalosporins [44].

#### 3.1.3. Diazabicyclooctanes (DBOs)

DBOs (Figure 4) are efficient β-lactamase inhibitors that are very potent against class A and class C β-lactamases. DBOs were first intended to be used as β-lactam mimics and it was found that they possess inhibition activity for β-lactamases and could be a source for β-lactamase inhibitors. Clavulanic acid is the only approved oral β-lactamase inhibitor and DBOs are none β-lactam β-lactamase inhibitors that have a sulfate group in their structure which limits their oral absorption [45]. DBOs have a five-membered ring with an amide group that targets the serine of the active site of the β-lactamase and forms a carbamoyl adduct [46].

Avibactam (NXL104, Figure 4) is a semi-synthetic compound approved by the FDA in 2014 as a combination therapy with ceftazidime to treat complicated intra-abdominal and complicated urinary tract infections [47]. Avibactam has excellent inhibitory activity against Ambler class A, class C, and some class D β-lactamases, but it was inefficient against OXA-23 and OXA-24, which are the main enzymes responsible for carbapenem-resistance in *A. baumannii* [48]. Two avibactam combinations are under clinical trials; ATM-AVI which is a combination of avibactam and aztreonam for the treatment of complicated intra-abdominal infections, and CXL in which avibactam is combined with ceftaroline fosamil (a prodrug for ceftaroline) for the treatment of multi-resistant bacterial infections [37,49]. Relebactam (MK-7655) (Figure 4) is another DBO β-lactamase inhibitor structurally related to avibactam. Relebactam is active against Gram-negative bacteria and is currently in phase 3 clinical trial in combination with imipenem/cilastatin for the treatment of drug-resistant infections including infections caused by carbapenem-resistant Enterobacteriaceae [49,50].

Zidebactam (Figure 4) is a bicyclo-acyl hydrazide DBO β-lactamase inhibitor. WCK5222, a combination of zebibactam and cefepime, is currently under clinical development (phase 1) for the treatment of serious infections caused by MDR Gram-negative pathogens including *P. aeruginosa* and *A. baumannii* [51].

#### 3.1.4. Boronic Acids as Transition State Analogs

Boronic acids have been recognized as inhibitors of serine proteases [52]. Diverse boronic acid analogues exhibited activity as inhibitors of β-lactamases of class A and C [53,54]. They inhibit the enzyme mainly by formation of tetrahedral intermediate with the catalytic serine residue, mimicking the transition state in the hydrolytic reaction that β-lactamases catalyze [52,54].

Many derivatives of boronic acid were synthesized and designed to have increased selectivity for β-lactamases over other serine proteases. The most promising analog was the compound RPX7009 (vaborbactam) (Figure 4), which is a cyclic boronate ester that restored the activity of carbapenems against KPC. RPX7009 combined with biapenem is called carbavance, and it is currently under phase 3 clinical trials for the treatment of various infections caused by KPC-producing *Enterobacteriaceae* [54,55].

### 3.2. Antibiotic Alternatives

#### 3.2.1. Bacteriophages

Bacteriophages or phages are bactericidal agents which are a type of virus that exclusively infect bacteria. Bacteriophages were discovered and named by Felix d’Herelle in 1917 [56]. The idea of using bacteriophages to treat infections is an old idea that has been overshadowed by antibiotic surge during the golden age of antibiotics. Today, after antibiotic resistance has reached a crisis, there is renewed interest in bacteriophage therapy. With advanced modern technology and genome sequencing availability, the discovery and development of new bacteriophage treatments are enjoying more interest.

Bacteriophages are bactericidals that disrupt many or all bacterial processes. Bacteriophages have a high degree of species or strain specificity, avoiding dysbacteriosis and secondary infections. Bacteriophages are unable to penetrate eukaryotic cells and no adverse effects have been reported so far. Phages are self-amplifying while target bacteria are present thus amplifying the local antibacterial effects. Unlike conventional antibiotics, phages can penetrate and destroy biofilms [57,58,59,60].

In contrast to antibiotics, new phages can be introduced in a much cheaper and faster process [57]. The obstacle is to perform properly designed, randomized, placebo-controlled, and double-blind clinical trials for the implementation of phage therapy. Most clinical trials have failed either to recruit enough patients or to provide statistically relevant conclusions. So far, there are only a few reports on the clinical use of bacteriophages [58,61]. 

In 2009, a controlled clinical study was conducted to evaluate the efficacy and safety of a new bacteriophage preparation called Biophage-PA, which is a cocktail of six phages with lytic activity against antibiotic-resistant *P. aeruginosa* in chronic otitis. No adverse events were reported indicating the safety of the therapy, and the trial showed efficacy as the count of *P. aeruginosa* was significantly reduced for the phage treated group compared to control [61].

A clinical study was conducted in 2016 to test the efficacy and safety of two phage cocktails against *E. coli* in acute diarrhea in children in Bangladesh. The phage administered was a T4-like coliphage containing 11 phages or a commercial Russian coliphage product 17 phage in oral rehydration solution. A control group was given the standard treatment of oral rehydration solution. There were no adverse effects observed but the phage cocktails did not improve diarrheal outcome over the control group. The reason for this might be that the phages failed to amplify in the intestine [62]. 

The most recent clinical trial of bacteriophages was a randomized, controlled, double-blind phase 1/2 trial to study the efficacy and tolerability of PhagoBurn (a cocktail of 12 natural lytic anti-*P. aeruginosa* bacteriophages in an alginate template) to treat burn wounds infected by *P. aeruginosa*. In this trial, PhagoBurn was added directly to the wound. The concentration of the phage cocktail had decreased after manufacturing and participants were given a lower concentration than expected. As a result, the phage cocktail decreased bacterial burden in burn wounds at a slower pace than the standard care given to the control group. The trial was stopped on Jan 2, 2017, due to insufficient efficacy [63].

Several in vitro and in vivo studies have reported synergism between bacteriophages and antibiotics. Oechslin et al. reported that an antipseudomonal phage cocktail was active and highly synergistic with ciprofloxacin both in vitro and in vivo, and it reduced virulence of *P. aeruginosa* and reduced bacterial load 10,000-fold in rats. in experimental endocarditis [64]. Huff et al. found that bacteriophage and the antibiotic enrofloxacin decreased the mortality of birds challenged with *E. coli* to no mortality compared to 3% and 15% mortality with individual treatments with enrofloxacin and bacteriophage, respectively [65]. 

However, the evolution of bacterial resistance to phages is unavoidable. Fortunately, bacteria resistant to one phage remain sensitive to other phages [57]. Despite all the potential of bacteriophage to help treat infections especially of resistant bacteria, some studies suggest that bacteriophages contribute to the evolution and spread of antibiotic resistance, as phages can be a vehicle for the acquisition, maintenance, and spread of antibiotic resistance genes [66].

#### 3.2.2. DCAP

The compound 2-((3-(3,6-dichloro-9*H*-carbazol-9-yl)-2-hydroxypropyl)amino)-2-(hydroxy-methyl)propane1,3-diol (DCAP, Figure 4) is an antibacterial agent introduced in 2012 by Hurley and colleagues. DCAP is a potent broad-spectrum antibiotic that kills Gram-positive and Gram-negative bacteria, including *E. coli* and *P. aeruginosa*. DCAP was discovered in a high throughput screen of small molecules that inhibit the activity of MipZ, an ATPase that regulates the placement of the division site in *Caulobacter crescentus* in vitro [67]. DCAP is a specific inhibitor of Gram-positive and Gram-negative bacteria membrane and has two mechanisms of action that lead to cell lysis first, by facilitating ion transport across the membrane and so decreases the membrane potential, and second, by disrupting the lipid bilayer permeability. It is suggested that the activity of DCAP on Gram-negative bacteria is due to the effect on the inner membrane [68,69].

DCAP is a membrane-active drug that has the advantage of acting against bacteria in the dormant phase and biofilms. And what makes it distinct from other membrane-active agents is the specificity toward bacterial membranes. DCAP does not affect the red blood cell membrane, and when tested for the effect on the viability of the mammalian cell; a decrease in the viability was observed only at high concentrations and after more than 6 h [68].

Hurley et al. performed a structure-activity relationship of DCAP by synthesizing 15 analogs and found that the stereochemistry of the compound does not affect the activity. In contrast, the aromaticity and electronegativity of the chlorine-substituted carbazole were found to be essential for the biological activity. According to this finding, they suggested that the orientation of DCAP in the membrane is via its planar and dipolar characteristics. The study also found that when the hydrophobicity of the tail region of DCAP increased, the antibiotic activity was enhanced. Hurley et al. also synthesized two promising analogs of DCAP with activity against *Bacillus anthrax* and *Francisella tularensis*. Co-dosing DCAP analogs with ampicillin or kanamycin (antibiotics that target the cell wall differently) showed synergistic antibiosis [69,70]. This indicates that DCAP and its analogs represent promising candidates for new antibiotic therapy for the treatment of persistent infections caused by slow-growing and dormant bacteria.

#### 3.2.3. Odilorhabdins (ODLs)

The ribosome is a major target for antibiotics, but due to increasing MDR bacteria, the effectiveness of ribosome targeting antibiotics is reduced, thus amplifying the need for the development of new compounds that can bind to novel sites on the ribosome [71]. Gram-positive *Actinomycetes* and Gram-negative *Xenrhabdus* are two bacterial genera known for their capacity to produce a great variety of secondary metabolites via possessing genes encoding for non-ribosomal peptide synthetases (NRPSs) and polyketide synthases (PKSs) [72].ODLs (Figure 4) are a new class of modified peptide antibiotics produced by enzymes of the NRPSs gene cluster of *Xenorhabdus nematophila*. ODLs have broad-spectrum antibacterial activity against Gram-positive and Gram-negative pathogens, including carbapenem-resistant *Enterobacteriaceae*. ODLs act as inhibitors by binding to the small subunit of bacterial ribosomes that are distinct from those of existing antibiotics, and make contact with rRNA and tRNA; which induce miscoding in the translation system and increase the affinity of non-cognate aminoacyl tRNAs to the ribosome. In vitro and in vivo studies show promising results that make this new class an attractive starting point to develop ODL clinical candidates [73].

#### 3.2.4. Peptidic benzimidazoles

The benzimidazole moiety is recognized as a pharmacophore of chemotherapeutic agents that act like antibacterial, antifungal, anthelmintic, antiviral, anticancer, and anti-inflammatory agents for the treatment of a diverse range of diseases [74,75]. Benzimidazole-containing molecules inhibit peptide deformylase (PDF). PDF is a metalloprotease that removes of the N-terminal formyl group from the first methionine of newly synthesized polypeptides through Fe^2+^ -mediated catalysis, PDF consequently stops ribosomal protein synthesis in bacteria, protozoans, and some fungi [76,77]. PDF represents a good target for antimicrobials because it is highly conserved and ubiquitous in bacteria, fungi, and protozoa, and at the same time it is not required by mammalian cells [78,79]. Most PDF inhibitors are pseudopeptidehydroxamic acids, peptidic benzimidazoles are emerging alternative inhibitors. Several peptidic benzimidazol conjugates have shown in vitro antimicrobial activities. Peptidic benzimidazol (Figure 4) can be promising novel antibiotics for resistant Gram-negative bacteria [45,79,80].

Buğday et al. have reported the synthesis for the first time of sixteen novel benzimidazole amino acid/dipeptide conjugates incorporating glycine, alanine, phenylalanine, cysteine, and glycine-glycine dipeptide substitutions at position 1 of the benzimidazoles. In vitro antibacterial and antifungal activities of the compounds were tested against Gram-positive bacteria (*S. aureus* and *E. faecium*NJ-1) and Gram-negative bacteria (*E. coli and P. aeruginosa*), *Candida albicans* and *Candida tropicalis*. The antioxidant activities were measured as well. All of the compounds tested showed low to moderate antimicrobial and antioxidant activities [80]. 

In 2018, Bird et al. reported concise and expedient syntheses of internal and C-terminal peptidic benzimidazoles. The reported method is simply performed in wholly solid-phase at room temperature with the need for only minimal purification [79]. The Bird et al. method is expected to be an essential tool for the design and synthesis of novel antibiotics incorporating the benzimidazole pharmacophore. 

#### 3.2.5. Quorum Sensing (QS) Inhibitors

Bacterial chemical communication (quorum sensing) is a term used to refer to coordinated bacterial gene expression to act as a population to regulate processes like virulence factor production, susceptibility to antibiotics and biofilm formation. QS is mediated by small molecules called auto-inducers (AIs) such as Autoinducer-2 (AI-2), the so-called universal auto-inducer, which is responsible for intra- and interspecies bacterial communication [81].The most common QS mediators in Gram-positive are the oligopeptides and in Gram-negative bacteria theN-acyl homoserine lactones (AHLs). (*S*)-4,5-Dihydroxy-2,3-pentanedione ((S)-DPD), is a QS modulator found in both Gram-negative and Gram-positive bacteria, which is phosphorylated to phosphoryl DPD by LsrK to activate QS. Therefore, DPD derivatives with different core structures can be used to inhibit LsrK and work as antimicrobial agents. Isobutyl-DPD and phenyl-DPD (Figure 4) showed activity to inhibit QS in combination with gentamicin and small molecules. 2-Substituted aminobenzoic acids are other compounds structurally related to DPD that were identified as LsrK inhibitors by virtual screening. Working on a hit-to-lead process to increase LsrK inhibition by changing in the heterocyclic core can be used as QS-interfering compounds in the treatment of bacterial infections [82]. Modulation or inhibition of QS has emerged as a potential therapeutic that can control several bacterial virulence factors such as biofilm formation and reduce the bad effect of bacterial infections. QS Inhibitors can be used in combination with other antibiotics to fight antimicrobial resistance [81].

#### 3.2.6. Metal-Based Antibacterial Agents

Metal complexes have been used extensively throughout recorded history in medicine [83,84]. In recent times, research into metal-based antimicrobials has become of great interest. Metal compounds have unique modes of action, and exist in a wider range of three-dimensional geometries, a feature that is associated with higher clinical success rates compared to the generally flat organic molecules [85,86]. There is intense ongoing research focused on metal-based drugs, and massive contribution to literature by such studies. Ruthenium, gallium, bismuth, silver and copper are the main metals that have been applied in metal-based antimicrobials.

Ruthenium (Ru): Ruthenium (Ru) complexes have significant biological activities due to their ability to bind to different targets in the cell, like nucleic acids and proteins. Ru(II) complexes also have photo-physical properties that can be used to study cellular accumulation and localization [87]. Not only Ru(II) complexes are used as imaging probes and anticancer agents, they have the potential to act as antimicrobial agents. Smitten et al. tested the effect of luminescent dinuclear Ru(II) complexes on *E. coli*. Luminescent derivatives showed potent activity against Gram-negative bacteria and some MDR strains. This activity is due to the disruption of bacterial cell wall, which was confirmed by membrane damage assays. These results may lead to vital new treatments to life-threatening Gram-negative and MDR bacteria [88]. Atakilt Abebe et al. tested the antibacterial activity in vitro for two complexes: ruthenium (III) ([Ru(phen)_2_Cl_2_]Cl·_2_H2O and [Ru(phen)_2_(G)Cl]2Cl·H_2_O) on Gram-positive (*S. aureus* and methicillin-resistant Staphylococcus aureus (MRSA)) and Gram-negative (*E. coli* and *K. pneumonia*) bacteria. Results showed that these complexes have a broad range of activities against these resistant bacteria more than Chloramphenicol and Ciprofloxacin. Therefore, Ru(II) complexes can be considered as a good candidate for antibiotic drug development [89].

Gallium (Ga): Gallium has been explored as an antimicrobial. Gallium compounds target bacterial iron uptake and/or iron metabolism by inhibiting iron-dependent enzymes. Due to its similarity to iron, Ga(III) is incorporated into iron-dependent enzymes, but cannot be reduced to Ga(II), inhibiting the enzyme. Gallium protoporphyrin IX showed antibacterial activity against Gram-negative *P. aeruginosa*, *K. pneumoniae* and *A. baumannii* and Gram-positive various strains [90]. Ga(NO3)3, a sources of free gallium is currently undergoing a phase 2 clinical trial as an intravenous treatment for chronic *P. aeruginosa* infections in patients with cystic fibrosis [91]. Recently, Wang et al. have reported the discovery of two subunits of RNA polymerase, RpoB and RpoC, as Ga-binding proteins in *P. aeruginosa*. They showed that gallium attenuates transcription, and that co-treatment of Ga(III) and acetate increases the efficacy for combating antibiotic resistant *P. aeruginosa* [92].

Bismuth (Bi): Bismuth has low toxicity to humans and is already present in some approved medications. Bismuth has however potent bacterial toxicity, and some bismuth compounds, like bismuth subsalicylate, colloidal bismuth subcitrate and ranitidine bismuth citrate, are used to treat *H. pylori* infections [93,94]. The mechanism of the bactericidal activity of bismuth complexes is still not well known, though, Wang et al. have shown that Bi(III) antimicrobial drugs inhibit broad spectrum metallo-β-lactamase; with one Bi(III) displacing two Zn(II) ions [95]. Bismuth(III) thiolates derived from small heterocyclic thiones showed potent broad spectrum antimicrobial activity against *Mycobacteriumsmegmatis*, *E. coli* and *Enterococcus faecalis* [96,97]. Bismuth phosphinates exhibited antibacterial activity against *E. coli*, and some Gram-positive pathogens like MRSA and vancomycin-resistant Enterococcus [98]. Moreover, a study showed that bismuth-containing quadruple therapy (bismuth subcitrate potassium, metronidazole, tetracycline and omeprazole) achieved effective eradication of metronidazole-resistant *H. pylori* in infected patient [99].

Silver (Ag): Silver, colloidal silver and silver nitrate have been applied as wound antiseptics long before the discovery of antibiotics [100]. Today, a range of silver-based compounds are still used for their medicinal properties, such as silver sulfadiazine, a broad-spectrum topical antibiotic for burn wounds [101,102]. Silver compounds display a broad spectrum of antibacterial activity and are often used as antibacterial additives [103,104]. The exact mechanism of action of silver compounds is largely unknown, most studies ascribed the antibacterial activity to silver ions (Ag(I)) being released through dissociative mechanisms inside of the bacteria [105]. 

Silver nanoparticles are known for their antibacterial activity in low concentrations against multidrug resistant strains of *P. aeruginosa*, *E. coli*, *Salmonella enterica*, and *Proteus mirabilis* [106,107,108,109]. N-Heterocyclic carbene–silver (Ag(I)-NHC) complexes are a new class of silver complexes that was found to have potent antimicrobial activity against numerous pathogens including antibiotic resistant bacteria [84,110,111]. Despite the promising potential of the new silver complexes, the clinical application has remained unattained goal, more investigation and research is required to improve selective uptake and accumulation and to understand the specific mode of action [111]. 

Copper (Cu): For centuries copper compounds have been used as disinfectant agents, but the urgent need for new antibiotics forced research to turn in a different direction to find alternative treatments, especially for compounds that have the ability to penetrate bacterial membranes. Copper plays a major roles in cellular processes in living organisms such as free radical control and oxidative phosphorylation. Despite this fact, copper ions were recognized in 2008 as a metallic antibacterial agent, since they cause inhibition of growth and have a toxic effect on bacteria at higher concentrations due to the production of reactive oxygen species [112,113,114,115]. Copper has been used in various forms to test its antimicrobial properties. Cu ions need specific transport proteins to cross membranes, which limits their penetration to the target. As a result, lipophilic ligands called Cu ionophores have been developed as Cu ion carriers to deliver Cu ions across bacterial membranes. Djoko et al. prepared copper (bis-thiosemicarbazone) (Cu(btsc)) complex and tested its antimicrobial activity against Gram-positive, and Gram-negative bacteria such as *N. gonorrhoeae*, *Mycobacterium tuberculosis*, and *E. coli,* and showed the susceptibility of these bacteria to Cu(btsc) complex and its ability to be used as treatment [116].In addition, copper has been found to act synergistically with drugs, complexes coupled with quinolone drugs have been studied and reported. Psomas et al. synthesized copper complexes with second generation quinolone norfloxacin and ofloxacin. Results showed that these complexes have similar or higher binding affinity to DNA than free quinolones and more data are needed to study their exact mechanism of action [117].On the other hand, copper was used in nanoparticles technologies and were found to have high antibacterial activity against Gram-positive and Gram-negative bacteria especially due to their ability to penetrate the defense layer of bacteria; the outer membrane [112,113]. However, development of copper resistance could become a serious concern in hospitals and environments. New studies have found that bacteria developed tolerance to copper ions and can protect themselves from the toxic effect by several mechanisms such as active efflux systems and detoxifications by copper oxidase enzymes [118].

## 4. Patient Education

The spread of resistant pathogenic bacteria such as Gram-negative bacteria represents a huge and emerging threat to public health, and this threat is increasing every day. The major reason for the low response or difficulties in treating multi-resistant Gram-negative bacteria is the excessive and overuse of antibiotics in community and in hospitals. Therefore, strategies must be planned and applied to decrease the use and unnecessary prescription of antibiotics. This can be achieved by: (1) educational lectures, campaigns or patient information leaflets which explain the misuse of antibiotics and the adverse effects, and (2) preventing self-medication by the control and registration of antibiotic drug consumption in pharmacies. This and other strategies can increase patient knowledge and decrease the improper use and prescription of antibiotics [8,119,120].

## 5. Conclusions

Resistant Gram-negative bacteria such as *Enterobacteriaceae*, *Pseudomonas aeruginosa, Acinetobacter baumannii*, *Salmonella* spp., *Neisseria gonorrhoeae*, *Haemophilus influenza, Campylobacter, Helicobacter pylori*, *and Shigella* spp. are a real threat and a burden on the health and economy, which is why the WHO has published a priority list for antibiotic-resistant bacteria to discover and develop new treatments urgently. Novel approaches are aiming to overcome intrinsic and acquired resistance of Gram-negative bacteria and represent hope for the future. Some treatments have succeeded in yielding activity against Gram-negative resistant bacteria by deactivating the mechanism of resistance like the action of the β-lactamase inhibitor antibiotic adjuvants. Another promising trend refers to Nature to develop naturally derived agents with antibacterial activity against novel targets, such as bacteriophages which are bactericidal viruses that disrupt many bacterial processes, in addition to ODLs, new modified peptide antibiotics that bind to small subunits of bacterial ribosomes and others like DCAP, peptidic benzimidazoles, QS inhibitors, and Ru complexes. On the other hand, overuse and the misuse of these drugs contribute the spread of antibiotic resistance globally, therefore, efforts must be done to educate people and implement new policies and control programs on how to use and dispense antibiotics. Gram-negative resistance bacteria are the most dangerous group among other MDR bacteria and all these new treatments and approaches are needed to limit antimicrobial resistance and MDR strains.

## References

[1] Coico R. (2006). Gram staining. Curr. Protoc. Microbiol..

[2] Moyes R.B., Reynolds J., Breakwell D.P. (2009). Differential Staining of Bacteria: Gram Stain. Curr. Protoc. Microbiol..

[3] Silhavy T.J., Kahne D., Walker S. (2010). The Bacterial Cell Envelope. Cold Spring Harb. Perspect. Boil..

[4] Murray P.R., Rosenthal K.S., Pfaller M.A. (2005). Medical Microbiology.

[5] Miller S.I. (2016). Antibiotic Resistance and Regulation of the Gram-Negative Bacterial Outer Membrane Barrier by Host Innate Immune Molecules. mBio.

[6] Datta P., Gupta V. (2019). Next-generation strategy for treating drug resistant bacteria: Antibiotic hybrids. Indian J. Med. Res..

[7] Exner M., Bhattacharya S., Christiansen B., Gebel J., Goroncy-Bermes P., Hartemann P., Heeg P., Ilschner C., Kramer A., Larson E. (2017). Antibiotic resistance: What is so special about multidrug-resistant Gram-negative bacteria?. GMS Hyg. Infect. Control..

[8] Oliveira J., Reygaert W.C. (2019). Gram Negative Bacteria.

[9] Ruppé E., Woerther P.-L., Barbier F. (2015). Mechanisms of antimicrobial resistance in Gram-negative bacilli. Ann. Intensiv. Care.

[10] Paterson D.L. (2006). Resistance in gram-negative bacteria: Enterobacteriaceae. Am. J. Med..

[11] Van Duin D. (2017). Carbapenem-resistant Enterobacteriaceae: What we know and what we need to know. Virulence.

[12] Lutgring J.D. (2019). Carbapenem-resistant Enterobacteriaceae: An emerging bacterial threat. Semin. Diagn. Pathol..

[13] Lee S.H., Lee J.H., Park M., Park K.S., Bae I.K., Kim Y.B., Cha C.-J., Jeong B.C., Lee S.H. (2017). Biology of Acinetobacter baumannii: Pathogenesis, Antibiotic Resistance Mechanisms, and Prospective Treatment Options. Front. Microbiol..

[14] Lin M.-F., Lan C.-Y. (2014). Antimicrobial resistance inAcinetobacter baumannii: From bench to bedside. World J. Clin. Cases.

[15] Pachori P., Gothalwal R., Gandhi P. (2019). Emergence of antibiotic resistance Pseudomonas aeruginosa in intensive care unit; a critical review. Gene Funct. Dis..

[16] Savoldi A., Carrara E., Graham D.Y., Conti M., Tacconelli E. (2018). Prevalence of Antibiotic Resistance in Helicobacter pylori: A Systematic Review and Meta-analysis in World Health Organization Regions. Gastroenterology.

[17] Park J., Dunbar K.B., Mitui M., Arnold C.A., Lam-Himlin R.M., Valasek M.A., Thung I., Okwara C., Coss E., Cryer B. (2016). Helicobacter pylori Clarithromycin Resistance and Treatment Failure Are Common in the USA. Dig. Dis. Sci..

[18] Alba C., Blanco A., Alarcón T. (2017). Antibiotic resistance in Helicobacter pylori. Curr. Opin. Infect. Dis..

[19] Smith J.L., Fratamico P.M. (2010). Fluoroquinolone Resistance in Campylobacter. J. Food Prot..

[20] Sproston E.L., Wimalarathna H.M.L., Sheppard S.K. (2018). Trends in fluoroquinolone resistance in Campylobacter. Microb. Genom..

[21] Cuypers W., Jacobs J., Wong V., Klemm E.J., Deborggraeve S., Van Puyvelde S. (2018). Fluoroquinolone resistance in Salmonella: Insights by whole-genome sequencing. Microb. Genom..

[22] A Crump J., Sjölund-Karlsson M., A Gordon M., Parry C.M. (2015). Epidemiology, Clinical Presentation, Laboratory Diagnosis, Antimicrobial Resistance, and Antimicrobial Management of Invasive Salmonella Infections. Clin. Microbiol. Rev..

[23] A Hill S., Masters T.L., Wachter J. (2016). Gonorrhea - an evolving disease of the new millennium. Microb. Cell.

[24] Bala M., Sood S. (2010). Cephalosporin Resistance in Neisseria gonorrhoeae. J. Glob. Infect. Dis..

[25] Barry P.M., Klausner J.D. (2009). The use of cephalosporins for gonorrhea: The impending problem of resistance. Expert Opin. Pharmacother..

[26] Ghanem K.G., Giles J.A., Zenilman J.M. (2005). Fluoroquinolone-resistant Neisseria gonorrhoeae: The Inevitable Epidemic. Infect. Dis. Clin. North Am..

[27] Lourenço A.P.R.D.C., Dos Santos K.T.B., Moreira B.M., Fracalanzza S.E.L., Bonelli R.R. (2017). Antimicrobial resistance in Neisseria gonorrhoeae: History, molecular mechanisms and epidemiological aspects of an emerging global threat. Braz. J. Microbiol..

[28] Heinz E. (2018). The return of Pfeiffer’s bacillus: Rising incidence of ampicillin resistance in *Haemophilus influenzae*. Microb. Genom..

[29] Tristram S., Jacobs M.R., Appelbaum P.C. (2007). Antimicrobial Resistance in Haemophilus influenzae. Clin. Microbiol. Rev..

[30] Bae S., Lee J., Lee J., Kim E., Lee S., Yu J., Kang Y. (2009). Antimicrobial Resistance in Haemophilus influenzae Respiratory Tract Isolates in Korea: Results of a Nationwide Acute Respiratory Infections Surveillance▿. Antimicrob. Agents Chemother..

[31] Qin T., Qian H., Fan W., Ma P., Zhou L., Dong C., Gu B., Huo X. (2017). Newest data on fluoroquinolone resistance mechanism of Shigella flexneri isolates in Jiangsu Province of China. Antimicrob. Resist. Infect. Control..

[32] Nüesch-Inderbinen M., Heini N., Zurfluh K., Althaus D., Hächler H., Stephan R. (2016). Shigella Antimicrobial Drug Resistance Mechanisms, 2004–2014. Emerg. Infect. Dis..

[33] Xu W.-C., Silverman M.H., Yu X.Y., Wright G., Brown N. (2019). Discovery and development of DNA polymerase IIIC inhibitors to treat Gram-positive infections. Bioorganic Med. Chem..

[34] Bernal P., Molina-Santiago C., Daddaoua A., Llamas M.A. (2013). Antibiotic adjuvants: Identification and clinical use. Microb. Biotechnol..

[35] Gill E.E., Franco O.L., Hancock R.E.W. (2014). Antibiotic Adjuvants: Diverse Strategies for Controlling Drug-Resistant Pathogens. Chem. Boil. Drug Des..

[36] Walsh C. (2000). Molecular mechanisms that confer antibacterial drug resistance. Nature.

[37] Gonzalez-Bello C. (2017). Antibiotic adjuvants – A strategy to unlock bacterial resistance to antibiotics. Bioorganic Med. Chem. Lett..

[38] Drawz S.M., Papp-Wallace K.M., Bonomo R.A. (2013). New β-Lactamase Inhibitors: A Therapeutic Renaissance in an MDR World. Antimicrob. Agents Chemother..

[39] Leonard D.A., Bonomo R.A., Powers R.A. (2013). Class D β-Lactamases: A Reappraisal after Five Decades. Accounts Chem. Res..

[40] Klingler F.-M., Wichelhaus T.A., Frank D., Cuesta-Bernal J., El-Delik J., Müller H.F., Sjuts H., Göttig S., Koenigs A., Pos K.M. (2015). Approved Drugs Containing Thiols as Inhibitors of Metallo-β-lactamases: Strategy To Combat Multidrug-Resistant Bacteria. J. Med. Chem..

[41] Reading C., Cole M. (1977). Clavulanic Acid: A Beta-Lactamase-Inhibiting Beta-Lactam from Streptomyces clavuligerus. Antimicrob. Agents Chemother..

[42] Drawz S.M., Bonomo R.A. (2010). Three Decades of β-Lactamase Inhibitors. Clin. Microbiol. Rev..

[43] Buynak J.D., Rao A., Doppalapudi V.R., Adam G., Petersen P.J., Nidamarthy S.D. (1999). The synthesis and evaluation of 6-alkylidene-2′β-substituted penam sulfones as β-lactamase inhibitors. Bioorganic Med. Chem. Lett..

[44] Vallejo J.A., Guitián M.M., Vázquez-Ucha J.C., Gonzalez-Bello C., Poza M., Buynak J.D., Bethel C.R., Bonomo R.A., Bou G., Beceiro A. (2016). LN-1-255, a penicillanic acid sulfone able to inhibit the class D carbapenemase OXA-48. J. Antimicrob. Chemother..

[45] Mangoni A.A., Guillou C., Vanden Eynde J.J., Hulme C., Jampilek J., Li W., Prokai-Tatrai K., Rautio J., Collina S., Tuccinardi T. (2018). Breakthroughs in Medicinal Chemistry: New Targets and Mechanisms, New Drugs, New Hopes (-)4. Molecules.

[46] Coleman K. (2011). Diazabicyclooctanes (DBOs): A potent new class of non-β-lactam β-lactamase inhibitors. Curr. Opin. Microbiol..

[47] Lagacé-Wiens P., Walkty A., A Karlowsky J. (2014). Ceftazidime–avibactam: An evidence-based review of its pharmacology and potential use in the treatment of Gram-negative bacterial infections. Core Évid..

[48] Vázquez-Ucha J.C., Maneiro M., Martínez-Guitián M., Buynak J., Bethel C.R., Bonomo R.A., Bou G., Poza M., Gonzalez-Bello C., Beceiro A. (2017). Activity of the β-Lactamase Inhibitor LN-1-255 against Carbapenem-Hydrolyzing Class D β-Lactamases from Acinetobacter baumannii. Antimicrob. Agents Chemother..

[49] Leone S., Damiani G., Pezone I., Kelly M.E., Cascella M., Alfieri A., Pace M.C., Fiore M. (2019). New antimicrobial options for the management of complicated intra-abdominal infections. Eur. J. Clin. Microbiol. Infect. Dis..

[50] Wright H., Bonomo R.A., Paterson D.L. (2017). New agents for the treatment of infections with Gram-negative bacteria: Restoring the miracle or false dawn?. Clin. Microbiol. Infect..

[51] Sader H.S., Castanheira M., Huband M., Jones R.N., Flamm R.K. (2017). WCK 5222 (Cefepime-Zidebactam) Antimicrobial Activity against Clinical Isolates of Gram-Negative Bacteria Collected Worldwide in 2015. Antimicrob. Agents Chemother..

[52] Smoum R., Rubinstein A., Dembitsky V.M., Srebnik M. (2012). Boron Containing Compounds as Protease Inhibitors. Chem. Rev..

[53] Beesley T., Gascoyne N., Knott-Hunziker V., Petursson S., Waley S.G., Jaurin B., Grundstrom T. (1983). The inhibition of class C β-lactamases by boronic acids. Biochem. J..

[54] Hecker S.J., Reddy K.R., Totrov M., Hirst G.C., Lomovskaya O., Griffith D.C., King P., Tsivkovski R., Sun N., Sabet M. (2015). Discovery of a Cyclic Boronic Acid β-Lactamase Inhibitor (RPX7009) with Utility vs Class A Serine Carbapenemases. J. Med. Chem..

[55] Goldstein E.J.C., Citron D.M., Tyrrell K.L., Merriam C.V. (2013). In Vitro Activity of Biapenem plus RPX7009, a Carbapenem Combined with a Serine β-Lactamase Inhibitor, against Anaerobic Bacteria. Antimicrob. Agents Chemother..

[56] D’Herelle F. (1917). Sur un microbe invisible antagoniste des bacilles dysentériques. CR Acad. Sci. Paris.

[57] Chanishvili N., Aminov R.I. (2019). Bacteriophage therapy: Coping with the growing antibiotic resistance problem. Microbiol. Aust..

[58] Kortright K.E., Chan B.K., Koff J.L., Turner P.E. (2019). Phage Therapy: A Renewed Approach to Combat Antibiotic-Resistant Bacteria. Cell Host Microbe.

[59] McCallin S., Alam Sarker S., Barretto C., Sultana S., Berger B., Huq S., Krause L., Bibiloni R., Schmitt B., Reuteler G. (2013). Safety analysis of a Russian phage cocktail: From MetaGenomic analysis to oral application in healthy human subjects. Virology.

[60] Międzybrodzki R., Borysowski J., Weber-Dąbrowska B., Fortuna W., Letkiewicz S., Szufnarowski K., Pawełczyk Z., Rogóż P., Kłak M., Wojtasik E. (2012). Clinical Aspects of Phage Therapy. Advances in Clinical Chemistry.

[61] Wright A., Hawkins C., Änggård E., Harper D. (2009). A controlled clinical trial of a therapeutic bacteriophage preparation in chronic otitis due to antibiotic-resistantPseudomonas aeruginosa; a preliminary report of efficacy. Clin. Otolaryngol..

[62] Alam Sarker S., Sultana S., Reuteler G., Moine D., Descombes P., Charton F., Bourdin G., McCallin S., Ngom-Bru C., Neville T. (2016). Oral Phage Therapy of Acute Bacterial Diarrhea With Two Coliphage Preparations: A Randomized Trial in Children From Bangladesh. EBioMedicine.

[63] Jault P., Leclerc T., Jennes S., Pirnay J.-P., Que Y.-A., Resch G., Rousseau A.F., Ravat F., Carsin H., Le Floch R. (2019). Efficacy and tolerability of a cocktail of bacteriophages to treat burn wounds infected by Pseudomonas aeruginosa (PhagoBurn): A randomised, controlled, double-blind phase 1/2 trial. Lancet Infect. Dis..

[64] Oechslin F., Piccardi P., Mancini S., Gabard J., Moreillon P., Entenza J.M., Resch G., Que Y.-A. (2017). Synergistic Interaction Between Phage Therapy and Antibiotics Clears Pseudomonas Aeruginosa Infection in Endocarditis and Reduces Virulence. J. Infect. Dis..

[65] Huff W.E., Huff G.R., Rath N.C., Balog J.M., Donoghue A.M. (2004). Therapeutic efficacy of bacteriophage and Baytril (enrofloxacin) individually and in combination to treat colibacillosis in broilers. Poult. Sci..

[66] Calero-Caceres W., Ye M., Balcázar J.L. (2019). Bacteriophages as Environmental Reservoirs of Antibiotic Resistance. Trends Microbiol..

[67] Thanbichler M., Shapiro L. (2006). MipZ, a Spatial Regulator Coordinating Chromosome Segregation with Cell Division in Caulobacter. Cell.

[68] Eun Y.J., Foss M.H., Kiekebusch D., Pauw D.A., Westler W.M., Thanbichler M., Weibel D. (2012). DCAP: A Broad-Spectrum Antibiotic That Targets the Cytoplasmic Membrane of Bacteria. J. Am. Chem. Soc..

[69] Heinrich V., Hurley K., Santos T., Weibel D. (2015). DCAP: A broad-spectrum antibiotic that targets the cytoplasmic membrane of bacteria. FASEB J..

[70] Hurley K.A., Heinrich V., Hershfield J.R., Demons S.T., Weibel D. (2015). Membrane-Targeting DCAP Analogues with Broad-Spectrum Antibiotic Activity against Pathogenic Bacteria. ACS Med. Chem. Lett..

[71] Polikanov Y.S., A Aleksashin N., Beckert B., Wilson D.N. (2018). The Mechanisms of Action of Ribosome-Targeting Peptide Antibiotics. Front. Mol. Biosci..

[72] Bérdy J. (2005). Bioactive Microbial Metabolites. J. Antibiot..

[73] Pantel L., Florin T., Dobosz-Bartoszek M., Racine E., Sarciaux M., Serri M., Houard J., Campagne J.-M., De Figueiredo R.M., Midrier C. (2018). Odilorhabdins, Antibacterial Agents that Cause Miscoding by Binding at a New Ribosomal Site. Mol. Cell.

[74] Bansal Y., Silakari O. (2012). The therapeutic journey of benzimidazoles: A review. Bioorganic Med. Chem..

[75] Boiani M., Gonzalez M. (2005). Imidazole and benzimidazole derivatives as chemotherapeutic agents. Mini-Reviews Med. Chem..

[76] Jain R., Chen D., White R.J., Patel D.V., Yuan Z. (2005). Bacterial Peptide deformylase inhibitors: A new class of antibacterial agents. Curr. Med. Chem..

[77] Becker A., Schlichting I., Kabsch W., Schultz S., Wagner A.F.V. (1998). Structure of Peptide Deformylase and Identification of the Substrate Binding Site. J. Boil. Chem..

[78] Nguyen K.T., Hu X., Colton C., Chakrabarti R., Zhu M.X., Pei D. (2003). Characterization of a Human Peptide Deformylase: Implications for Antibacterial Drug Design†. Biochemistry.

[79] Bird M., Silvestri A.P., Dawson P.E. (2018). Expedient on-resin synthesis of peptidic benzimidazoles. Bioorganic Med. Chem. Lett..

[80] Bugday N., Kucukbay F.Z., Apohan E., Küçükbay H., Serindag A., Yesilada O. (2017). Synthesis and Evaluation of Novel Benzimidazole Conjugates Incorporating Amino Acids and Dipeptide Moieties. Lett. Org. Chem..

[81] Stotani S., Gatta V., Medda F., Padmanaban M., Karawajczyk A., Tammela P., Giordanetto F., Tzalis D., Collina S. (2018). A Versatile Strategy for the Synthesis of 4,5-Dihydroxy-2,3-Pentanedione (DPD) and Related Compounds as Potential Modulators of Bacterial Quorum Sensing. Molecules.

[82] Stotani S., Gatta V., Medarametla P., Padmanaban M., Karawajczyk A., Giordanetto F., Tammela P., Laitinen T., Poso A., Tzalis D. (2019). DPD-Inspired Discovery of Novel LsrK Kinase Inhibitors: An Opportunity To Fight Antimicrobial Resistance. J. Med. Chem..

[83] Gasser G. (2015). Metal Complexes and Medicine: A Successful Combination. Chim. Int. J. Chem..

[84] Medici S., Peana M.F., Crisponi G., Nurchi V.M., Lachowicz J.I.I., Remelli M., Zoroddu M.A. (2016). Silver coordination compounds: A new horizon in medicine. Co-ord. Chem. Rev..

[85] Lovering F., Bikker J., Humblet C. (2009). Escape from Flatland: Increasing Saturation as an Approach to Improving Clinical Success. J. Med. Chem..

[86] Hung A.W., Ramek A., Wang Y., Kaya T., Wilson J.A., Clemons P.A., Young D.W. (2011). Route to three-dimensional fragments using diversity-oriented synthesis. Proc. Natl. Acad. Sci..

[87] Li F., Collins J.G., Keene F.R. (2015). Ruthenium complexes as antimicrobial agents. Chem. Soc. Rev..

[88] Smitten K.L., Southam H.M., De La Serna J.B., Gill M.R., Jarman P., Smythe C., Poole R.K., Thomas J.A. (2019). Using Nanoscopy To Probe the Biological Activity of Antimicrobial Leads That Display Potent Activity against Pathogenic, Multidrug Resistant, Gram-Negative Bacteria. ACS Nano.

[89] Abebe A., Hailemariam T. (2016). Synthesis and Assessment of Antibacterial Activities of Ruthenium(III) Mixed Ligand Complexes Containing 1,10-Phenanthroline and Guanide. Bioinorg. Chem. Appl..

[90] Bonchi C., Imperi F., Minandri F., Visca P., Frangipani E. (2014). Repurposing of gallium-based drugs for antibacterial therapy. BioFactors.

[91] A Phase 2 IV Gallium Study for Patients With Cystic Fibrosis (IGNITE Study). https://clinicaltrials.gov/ct2/show/NCT02354859.

[92] Wang Y., Han B., Xie Y., Wang H., Wang R., Xia W., Li H., Sun H. (2019). Combination of gallium(iii) with acetate for combating antibiotic resistant Pseudomonas aeruginosa. Chem. Sci..

[93] Fock K.M., Graham D.Y., Malfertheiner P. (2013). Helicobacter pylori research: Historical insights and future directions. Nat. Rev. Gastroenterol. Hepatol..

[94] Li H., Wang R., Sun H. (2018). Systems Approaches for Unveiling the Mechanism of Action of Bismuth Drugs: New Medicinal Applications beyond Helicobacter Pylori Infection. Accounts Chem. Res..

[95] Wang R., Lai T.-P., Gao P., Zhang H., Ho P.-L., Woo P.C.-Y., Ma G., Kao R.Y., Li H., Sun H. (2018). Bismuth antimicrobial drugs serve as broad-spectrum metallo-β-lactamase inhibitors. Nat. Commun..

[96] Luqman A., Blair V.L., Brammananth R., Crellin P.K., Coppel R.L., Andrews P.C. (2014). Homo- and Heteroleptic Bismuth(III/V) Thiolates from N-Heterocyclic Thiones: Synthesis, Structure and Anti-Microbial Activity. Chem. A Eur. J..

[97] Luqman A., Blair V.L., Brammananth R., Crellin P.K., Coppel R.L., Andrews P.C. (2016). The Importance of Heterolepticity in Improving the Antibacterial Activity of Bismuth(III) Thiolates. Eur. J. Inorg. Chem..

[98] Werrett M.V., Herdman M.E., Brammananth R., Garusinghe U., Batchelor W., Crellin P.K., Coppel R.L., Andrews P.C. (2018). Bismuth Phosphinates in Bi-Nanocellulose Composites and their Efficacy towards Multi-Drug Resistant Bacteria. Chem. A Eur. J..

[99] Muller N., Amiot A., Le Thuaut A., Bastuji-Garin S., Deforges L., Delchier J.C. (2016). Rescue therapy with bismuth-containing quadruple therapy in patients infected with metronidazole-resistant Helicobacter pylori strains. Clin. Res. Hepatol. Gastroenterol..

[100] Alexander J.W. (2009). History of the Medical Use of Silver. Surg. Infect..

[101] Aziz Z., Abu S., Chong N.J. (2012). A systematic review of silver-containing dressings and topical silver agents (used with dressings) for burn wounds. Burns.

[102] Sierra M.A., Casarrubios L., La Torre M.C. (2019). Bio-Organometallic Derivatives of Antibacterial Drugs. Chem. A Eur. J..

[103] Yan J., AbdelGawad A., Rojas O.J., El-Naggar M. (2016). Antibacterial activity of silver nanoparticles synthesized In-situ by solution spraying onto cellulose. Carbohydr. Polym..

[104] Mijnendonckx K., Leys N., Mahillon J., Silver S., Van Houdt R. (2013). Antimicrobial silver: Uses, toxicity and potential for resistance. BioMetals.

[105] Frei A. (2020). Metal Complexes, an Untapped Source of Antibiotic Potential?. Antibiotics.

[106] Ma Y., Liu C., Qu D., Chen Y., Huang M., Liu Y. (2017). Antibacterial evaluation of sliver nanoparticles synthesized by polysaccharides from Astragalus membranaceus roots. Biomed. Pharmacother..

[107] Al-Hamadani A.H., Kareem A.A. (2017). Combination effect of edible mushroom–sliver nanoparticles and antibiotics against selected multidrug biofilm pathogens. Iraq Med. J..

[108] Kareem A. (2018). Combination Effect of Edible Mushroom – Sliver Nanoparticles and Antibioticsagainst selected Multidrug Biofilm Pathogens. Int. J. Res. Pharm. Sci..

[109] Rai M., Deshmukh S., Ingle A., Gade A. (2012). Silver nanoparticles: The powerful nanoweapon against multidrug-resistant bacteria. J. Appl. Microbiol..

[110] Kascatan-Nebioglu A., Panzner M.J., A Tessier C., Cannon C.L., Youngs W.J. (2007). N-Heterocyclic carbene–silver complexes: A new class of antibiotics. Co-ord. Chem. Rev..

[111] Johnson N.A., Southerland M.R., Youngs W.J. (2017). Recent Developments in the Medicinal Applications of Silver-NHC Complexes and Imidazolium Salts. Molecules.

[112] Vincent M., Hartemann P., Engels-Deutsch M. (2016). Antimicrobial applications of copper. Int. J. Hyg. Environ. Heal..

[113] Vincent M., Duval R., Hartemann P., Engels-Deutsch M. (2018). Contact killing and antimicrobial properties of copper. J. Appl. Microbiol..

[114] Dalecki A.G., Crawford C.L., Wolschendorf F. (2017). Copper and Antibiotics. Adv. Microb. Physiol..

[115] Bondarczuk K., Piotrowska-Seget Z. (2013). Molecular basis of active copper resistance mechanisms in Gram-negative bacteria. Cell Boil. Toxicol..

[116] Djoko K., Goytia M.M., Donnelly P.S., A Schembri M., Shafer W.M., McEwan A. (2015). Copper(II)-Bis(Thiosemicarbazonato) Complexes as Antibacterial Agents: Insights into Their Mode of Action and Potential as Therapeutics. Antimicrob. Agents Chemother..

[117] Živec P., Perdih F., Turel I., Giester G., Psomas G. (2012). Different types of copper complexes with the quinolone antimicrobial drugs ofloxacin and norfloxacin: Structure, DNA- and albumin-binding. J. Inorg. Biochem..

[118] Thummeepak R., Pooalai R., Harrison C., Gannon L., Thanwisai A., Chantratita N., Millard A., Sitthisak S. (2020). Essential Gene Clusters Involved in Copper Tolerance Identified in Acinetobacter baumannii Clinical and Environmental Isolates. Pathogens.

[119] Lee C.-R., Lee J.H., Kang L.-W., Jeong B.C., Lee S.H. (2015). Educational Effectiveness, Target, and Content for Prudent Antibiotic Use. BioMed Res. Int..

[120] De Bont E.G.P.M., Alink M., Falkenberg F.C.J., Dinant G.-J., Cals J. (2015). Patient information leaflets to reduce antibiotic use and reconsultation rates in general practice: A systematic review. BMJ Open.

